# Exploring the Potential of IL-4 and IL-13 Plasma Levels as Biomarkers in Atopic Dermatitis

**DOI:** 10.3390/life14030352

**Published:** 2024-03-07

**Authors:** George G. Mitroi, Elena Leocadia Pleșea, George F. Mitroi, Mihaela Roxana Mitroi, Carmen Daniela Neagoe, Simona Laura Ianoși

**Affiliations:** 1 Department of Dermatology, Faculty of Medicine, University of Medicine and Pharmacy of Craiova, 200349 Craiova, Romania; 2 Department of Bacteriology, Virology and Parasitology, University of Medicine and Pharmacy of Craiova, 200349 Craiova, Romania; 3 Department of Urology, Faculty of Medicine, University of Medicine and Pharmacy of Craiova, 200349 Craiova, Romania; 4 Department of Otorhinolaryngology, Faculty of Medicine, University of Medicine and Pharmacy of Craiova, 200349 Craiova, Romania; 5 Department of Internal Medicine, Faculty of Medicine, University of Medicine and Pharmacy of Craiova, 200349 Craiova, Romania

**Keywords:** atopic dermatitis, dupilumab, interleukin-4, interleukin-13, biomarker, serum levels

## Abstract

Atopic dermatitis (AD) is a persistent inflammatory skin condition that impacts individuals of various age groups, including both children and adults. Its pathophysiology involves allergens penetrating a disrupted epidermal barrier, triggering the dermal cells to produce pro-inflammatory cytokines and eliciting a T-cell-mediated immune response. Notably, interleukins (ILs), particularly interleukin 4 (IL-4) and interleukin 13 (IL-13), play a key role in AD pathogenesis. Therapies directed at inflammatory mechanisms, including Dupilumab, have demonstrated notable effectiveness in enhancing skin lesions, alleviating subjective symptoms, and improving the overall quality of life for individuals with AD. Despite therapeutic advances, assessing AD severity remains challenging. The commonly used tools, such as the SCORAD and DLQI scores, rely on subjective patient responses. Paraclinically, the search for universal biomarkers continues, with efforts to identify reliable indicators reflecting disease severity and treatment response. Various biomarkers, including Th2-related chemokines and cytokines, have been explored, but none have gained universal recognition for routine clinical use. This study aims to investigate the dynamics of the plasma levels of IL-4 and IL-13 during Dupilumab treatment and establish correlations between these ILs and disease severity, as measured using the SCORAD and DLQI scores. The ultimate endpoint is to determine whether IL-4 and IL-13 can serve as reliable biomarkers, assessing their correlation with patient-reported feelings and disease activity and potentially influencing their inclusion or exclusion as diagnostic elements in routine clinical practice.

## 1. Introduction

Atopic dermatitis (AD) is a persistent, itching inflammatory skin condition that impacts around 25% of children and 2–3% of adults [1]. One recent study encompassing 20,012 adults in France highlighted AD as the predominant chronic skin inflammatory condition, with a prevalence of 4.65%, surpassing other dermatological conditions such as psoriasis, alopecia areata, vitiligo, and hidradenitis suppurativa [2]. Summarily, the pathophysiology of AD begins with allergens penetrating the disrupted epidermal barrier. In response, the dermal cells are triggered to produce pro-inflammatory cytokines, which produces a T-cell-mediated immune response. AD onset is characterized by increases in T helper cells type 2 (Th2), such as IL-4, IL-5, IL-13, and IL-31, and T helper cells type 22 (Th22), such as IL-22 [3]. Among the Th2 immune mediators, IL-4 and IL-13 have been demonstrated to play a key role in AD pathogenesis [4]. Recent progress in understanding the pathophysiology of this disease has allowed the development of therapies aimed at the inflammatory mechanisms that initiate the condition. The first biological treatment was approved in 2017 and revolutionized the strictly symptomatic treatment used until that moment. Dupilumab, a humanized monoclonal antibody, selectively targets the α-subunit of the IL-4 receptor, a constituent of the IL-4 and IL-13 receptor complex. Regarding IL-4 and IL-13, it is crucial to highlight their distinct roles, IL-4 in Th2 polarization and IL-13 as an effector cytokine. Emphasizing the potential differences arising between simultaneous blockage of both and selective IL-13 blockage is essential. For instance, while eosinophilia [5] and psoriasis [6] are common side effects of Dupilumab, they are rarely observed with single IL-13 blockers. Numerous studies have consistently indicated favorable outcomes in patients subjected to Dupilumab treatment, showcasing its substantial efficacy in ameliorating skin lesions associated with atopic dermatitis (AD), alleviating subjective symptoms, enhancing overall quality of life, and mitigating the symptoms of anxiety and depression among individuals with AD [7,8]. However, the clinical tools most frequently used to assess the severity of AD are the Scoring Atopic Dermatitis (SCORAD) score and the Dermatology Life Quality Index (DLQI) score. These questionnaires are quite relative because they quantify subjective symptoms and therefore can be influenced by a patient’s individual perception of the disease. We recognize the importance of objectivity in our study and acknowledge the relevance of incorporating additional tools such as the Patient-Oriented Eczema Measure (POEM) and the Atopic-Dermatitis-Specific Quality of Life (ABS-A) scale [9]. Notably, ABS-A, unlike the DLQI, offers the advantage of being specifically designed to assess the impact of atopic dermatitis on quality of life. Nevertheless, we opted for the DLQI and SCORAD scores as assessment tools due to their extensive global utilization, providing a widely recognized benchmark. While acknowledging the relevance of the POEM and ABS-A, the preference for the DLQI and SCORAD scores ensures alignment with the prevailing international standards for a more universally comparable evaluation in our study.

Paraclinically, there is still no universal biomarker accepted and used in current practice. While AD stands as the most prevalent chronic inflammatory skin disease, endeavors are underway to ascertain dependable biomarkers capable of indicating the disease’s severity and response to treatment and serving as a distinctive and reliable diagnostic component. Recent technological advances and the emergence of new methods of analysis such as transcriptomics, genomics, proteomics, and deep next-generation sequencing can be used as tools to find potential biomarkers [10,11,12,13].

The term “biomarker” is defined by the Food and Drug Administration (FDA) as “a defined characteristic that is measured as an indicator of normal biological processes, pathogenic processes, or responses to an exposure or intervention, including therapeutic interventions. Molecular, histologic, radiographic, or physiologic characteristics are types of biomarkers. A biomarker is not an assessment of how a patient feels, functions, or survives” [14].

A considerable array of biomarkers have been identified and clinically employed to evaluate the severity of AD or gauge the treatment outcomes. The most frequently used biomarkers include Th2-related chemokines, namely macrophage-derived chemokine (MDC/CCL22), pulmonary and activation-regulated chemokine (PARC/CCL18), cutaneous T-cell-attracting chemokine (CTACK/CCL27), eosinophil-attracting chemokine (eotaxin-3/CCL26), the key Th2 cytokine IL-13, and the key Th22 cytokine IL-22 [15]. However, as of yet, none of these biomarkers have achieved universal recognition for incorporation into routine clinical practice and the management of atopic dermatitis (AD) [16].

In the present study, we aimed to measure the dynamics of the plasma levels of IL-4 and IL-13 during treatment with Dupilumab, the key immune mediators in AD pathogenesis. We also aimed to establish a correlation between the IL serum levels and the severity of the disease, measured using the two severity scores mentioned earlier, SCORAD and the DLQI. Therefore, we want to establish whether these ILs correlate with how a patient feels and with the activity and severity of the disease and thus assess the introduction or exclusion of IL-4 and IL-13 as a reliable biomarker.

## 2. Materials and Methods

### 2.1. Recruitment of the Study Participants

A total of 22 individuals with clinically diagnosed atopic dermatitis (AD) and 20 healthy volunteers were included in the case–control study. The diagnosis of AD was based on the modified Hanifin and Rajka criteria [17].

The inclusion criteria for patients with atopic dermatitis (AD) were as follows: (1) a confirmed diagnosis of AD for a minimum of 2 years, (2) age exceeding 18 years, (3) no prior receipt of biologic treatments, (4) absence of other dermatologic or systemic comorbidities, and (5) manifestation of severe AD as assessed using SCORAD. Additionally, twenty healthy volunteers with no history of dermatological or atopic conditions were incorporated into the study. This research received approval from the Ethics Committee of the University of Medicine and Pharmacy of Craiova (No. 182/1 November 2021), and all the participants provided written informed consent.

### 2.2. Collection of the Clinical and Laboratory Data

The clinical characteristics of the subjects, including age, gender, and the duration of AD, were documented. Severity scores evaluated using the DLQI and SCORAD were acquired from all AD patients both at the outset and during the course of treatment with Dupilumab. The participants were divided into two groups: the control group and the AD patient group.

### 2.3. Treatment of the AD Patients

Among the AD patients enrolled, participants were treated with Dupilumab at a loading dose of 600 mg followed by a 300 mg maintenance dose once every two weeks along with topical emollients. The clinical and laboratory data of the subjects were gathered at the baseline and week 12, in accordance with the timetable recommended by the drug producer for the assessment of therapeutic safety and clinical effectiveness [18].

### 2.4. Measurement of the Serum IL-4 and IL-13 Levels

Blood samples were obtained from both the patients and healthy subjects. In addition to conventional blood sampling methods, 3 mL blood samples were collected using 5 mL BD biochemistry vacutainers with separator gel and D-vac clot activator. Plastic sterile tubes with separator gel and clot serum separation tubes designed for enzyme-linked immunosorbent assays (ELISAs) were also utilized. The serum levels of IL-13 and IL-4 were determined using two commercial ELISA kits from DRG Instruments GmbH, Marburg, Germany—EIA 4841 for IL-13 and EIA 5539 for IL-4, respectively. The resulting color reaction was measured, according to the manufacturer’s instructions, at an absorbance of 450 nm using a Stat Fax ChroMate 4300 (Awareness Technology, Palm City, FL, USA) semi-automated analyzer/microplate reader.

### 2.5. Statistical Analysis

Data are expressed as mean ± standard deviation (SD). The statistical analyses were performed using Microsoft Excel for Mac, version 16.82 (Microsoft Corporation, Redmond, WA, USA). Normality was satisfied in each group of subjects (control group and AD patients). Statistical analyses were conducted using Student’s two-sample *t*-tests for independent samples and paired *t*-tests (to assess the differences before vs. after treatment in the AD patient group). Correlations were calculated using Pearson’s correlation analysis. A *p* value of less than 0.05 was considered statistically significant.

## 3. Results

### 3.1. Characteristics of the Study Subjects

Table 1 outlines the general characteristics of the study participants. Among the total 42 subjects, there were 22 males (52.38%) and 20 females (47.62%), with a mean age of 31.96 ± 3.69 years. The distribution of sexes and ages exhibited similarities between the control and atopic dermatitis (AD) groups.

### 3.2. IL-4 and IL-13 Levels Were Higher in the AD Group

**Figure 1:**
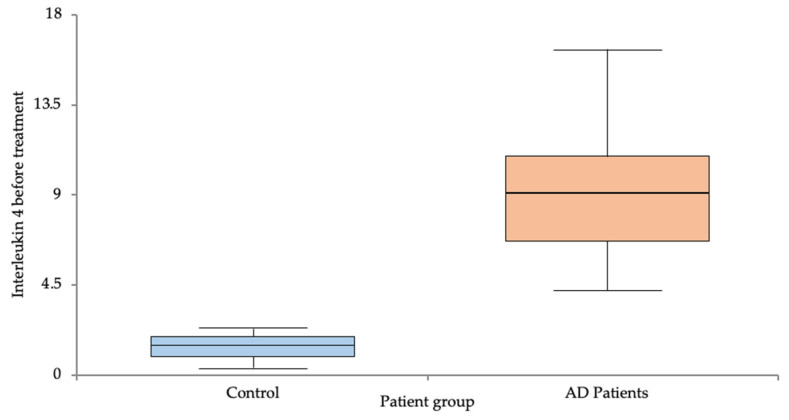
IL-4 levels pre-treatment in the control vs. AD groups.

**Figure 2:**
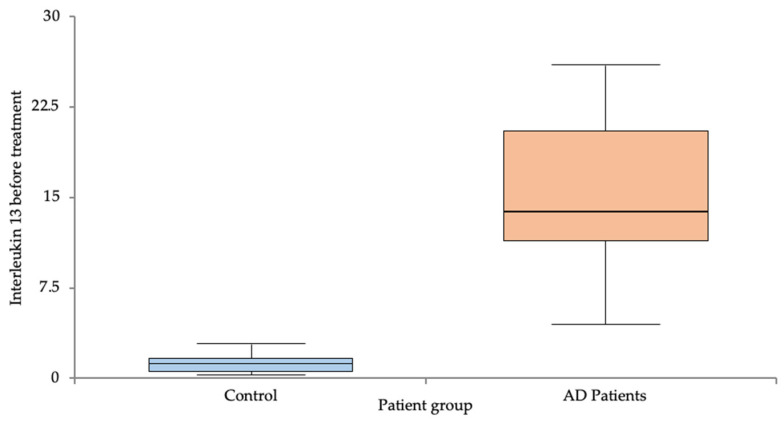
IL-13 levels pre-treatment in the control vs. AD groups.

The IL-4 levels were significantly lower in the control group compared to the AD group (1.42 ± 0.61 vs. 9.51 ± 3.71, *p* < 0.001) (Figure 1). Also, the IL-13 levels were significantly lower in the control group compared to the AD group (1.25 ± 0.78 vs. 15.55 ± 6.34, *p* < 0.001) (Figure 2) before treatment with Dupilumab. The persistent elevation in IL values relative to the control group continued following the implementation of the treatment, as evidenced by the post-treatment increase, as shown below. The results are summarized in Table 2.

### 3.3. Association of IL-4 Levels with SCORAD and the DLQI before Dupilumab Treatment in the AD Group

**Figure 3:**
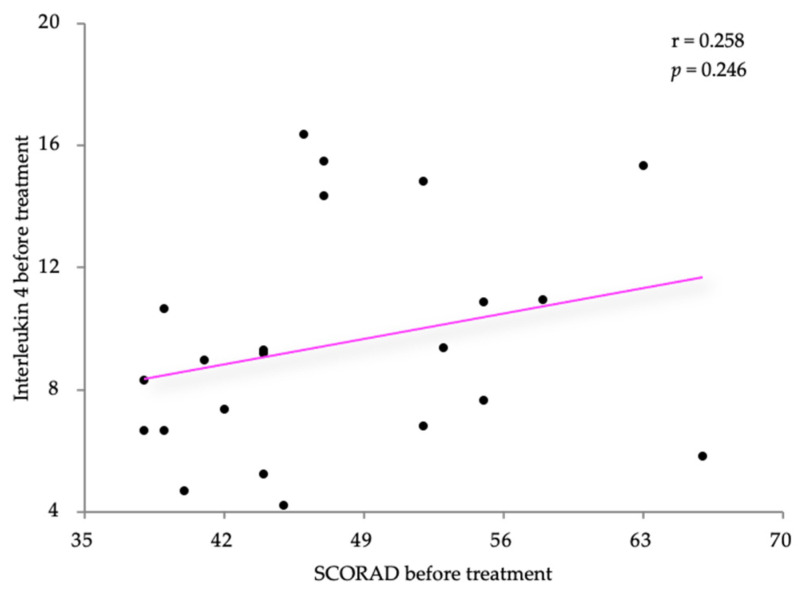
Correlation between IL-4 levels and SCORAD, before treatment.

**Figure 4:**
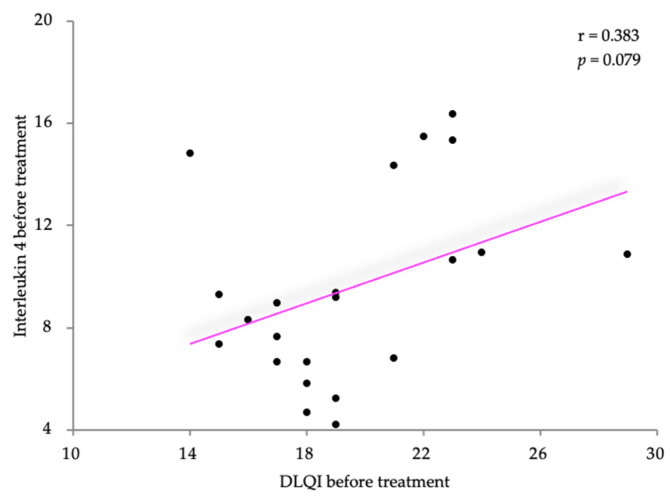
Correlation between IL-4 levels and DLQI, before treatment.

The associations between the clinical and laboratory values of the IL-4 levels in the atopic dermatitis (AD) patients were examined using Pearson’s correlation coefficient. The IL-4 levels exhibited a slight positive correlation with the SCORAD scores (r = 0.258) but lacked statistical significance (*p* = 0.246). The results are shown in Figure 3. Similar results were obtained for the correlation between the IL-4 levels and the DLQI score (Figure 4). The IL-4 levels were moderately positively correlated with the SCORAD scores (r = 0.383) but also without statistical significance (*p* = 0.079).

### 3.4. Association of the IL-13 Levels with SCORAD and the DLQI before Dupilumab Treatment in the AD Group

**Figure 5:**
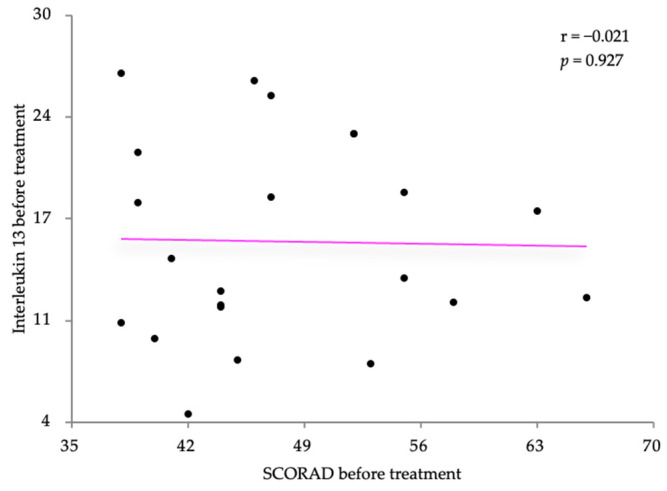
Correlation between IL-13 levels and SCORAD, before treatment.

**Figure 6:**
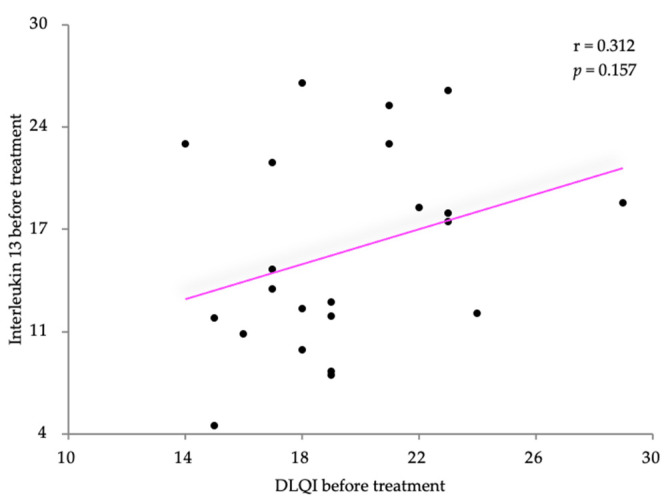
Correlation between IL-13 levels and DLQI, before treatment.

Pearson’s correlation coefficient was also used to analyze the relationship between IL-13 and the two disease severity scores, SCORAD and the DLQI. The IL-13 levels were slightly negatively correlated with the SCORAD scores (r = −0.021) with no statistical significance (*p* = 0.927) (Figure 5). Relatively similar results were also obtained regarding the correlation with the DLQI score (r = 0.312, *p* = 0.157) (Figure 6).

### 3.5. The Values of Both IL-4 and IL-13 Were Higher after Treatment

**Figure 7:**
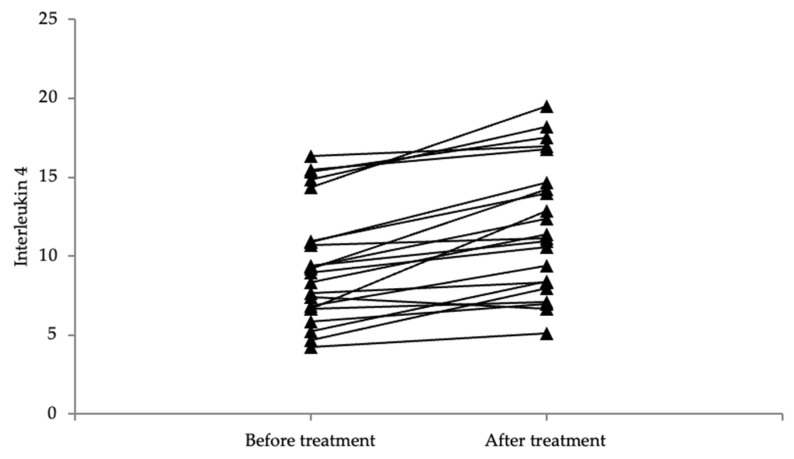
Graphical representation of IL-4 values before and after treatment.

**Figure 8:**
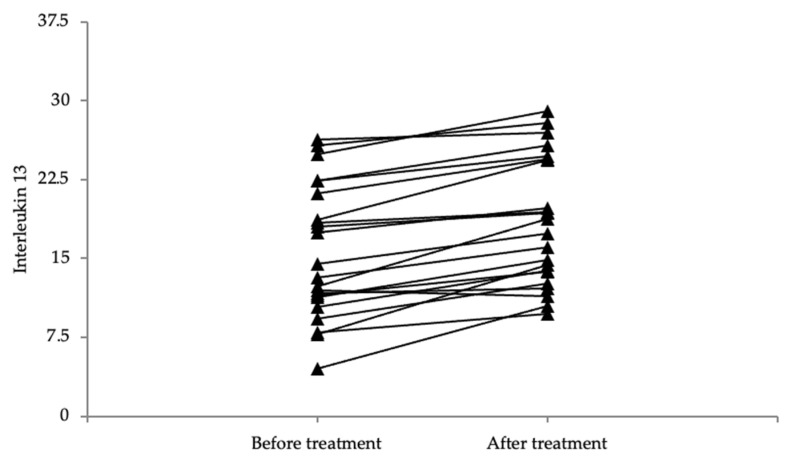
Graphical representation of IL-13 values before and after treatment.

The values of both IL-4 and IL-13 are significantly higher following treatment as compared to the values before treatment (*p* < 0.001) (Figure 7 and Figure 8). The difference in SCORAD and the DLQI before vs. after treatment is even greater, with the scores being up to 7 times lower following treatment as compared to the initial scores, marking a statistically significant improvement in the SCORAD and DLQI scores (*p* < 0.001) (Table 3).

### 3.6. Association of the IL-4 Levels with SCORAD and the DLQI after Dupilumab Treatment in the AD Group

**Figure 9:**
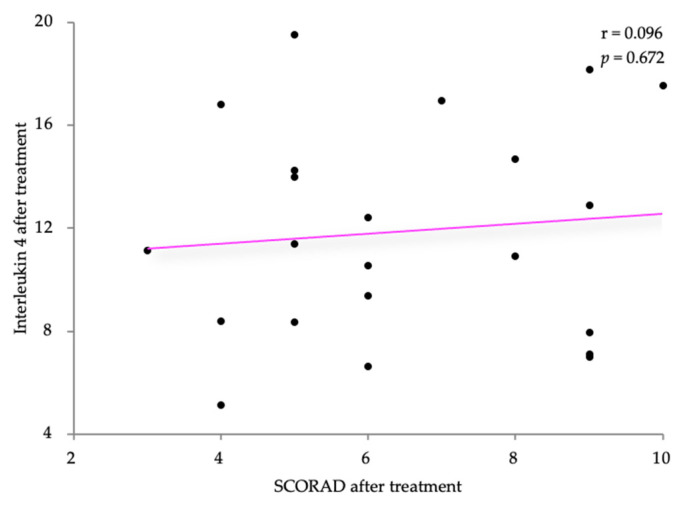
Correlation between IL-4 levels and SCORAD, after treatment.

**Figure 10:**
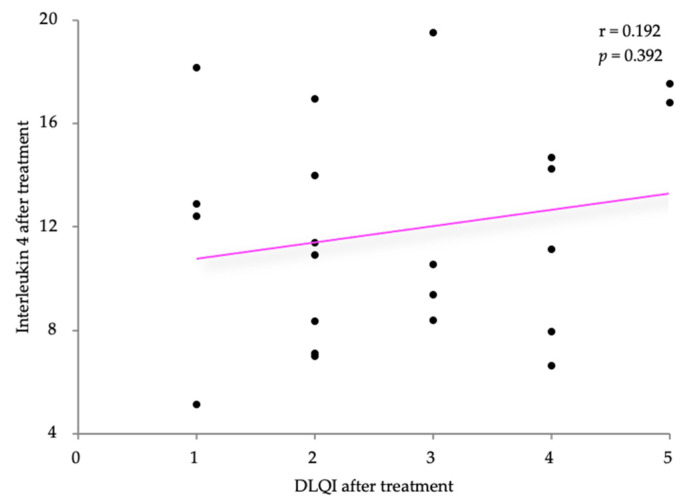
Correlation between IL-5 levels and DLQI, after treatment.

Using Pearson’s correlation coefficient, we analyzed the relationship between the IL-4 levels following treatment with the SCORAD and DLQI scores after treatment in the AD patients. The values are weakly positively correlated (r = 0.096 and r = 0.192, respectively), without statistical significance (*p* = 0.672 and *p* = 0.392, respectively). The results are shown in Figure 9 and Figure 10.

### 3.7. Association of the IL-4 Levels with SCORAD and the DLQI after Dupilumab Treatment in the AD Group

**Figure 11:**
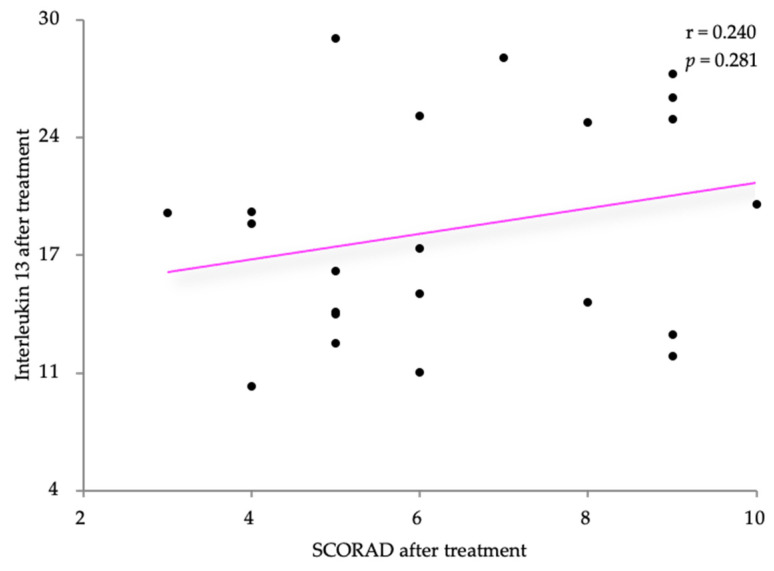
Correlation between IL-13 levels and SCORAD, after treatment.

**Figure 12:**
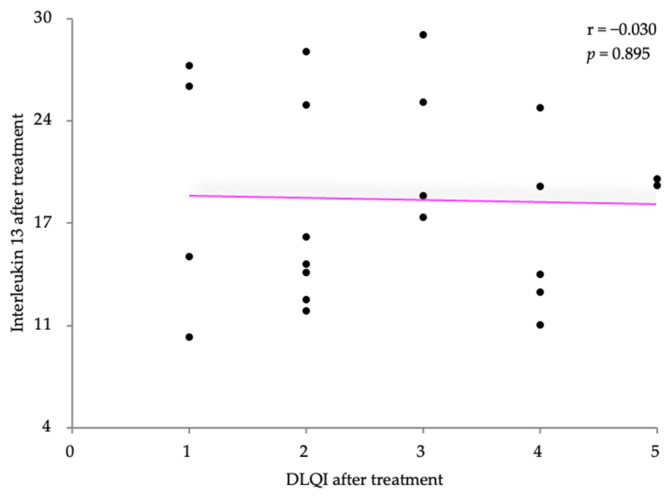
Correlation between IL-13 levels and DLQI, after treatment.

Pearson’s correlation coefficient was also used to analyze the relationship after treatment between IL-13 and the two disease severity scores, SCORAD and the DLQI. The IL-13 levels were slightly positively correlated with the SCORAD scores (r = 0.240) with no statistical significance (*p* = 0.281) (Figure 11). A very weak negative correlation was observed after treatment between the IL-13 levels and the DLQI score (r = −0.030, *p* = 0.895) (Figure 12).

## 4. Discussion

### 4.1. Cross-Analysis

In our investigation, we conducted a clinical assessment of IL-4 and IL-13 as potential biomarkers for disease severity and the treatment response in atopic dermatitis (AD). However, it is important to acknowledge certain limitations within this study. Initially, our observation did not indicate a reduction in serum IL-4 and IL-13 levels in the successfully treated patients, underscoring the requirement for further long-term prospective case–control studies. Secondly, to establish IL-4 and IL-13 as novel biomarkers, a subsequent validation study employing immunohistochemical analysis of patients’ lesional and non-lesional skin is imperative. Thirdly, the validation of IL-4 and IL-13 was limited by the sample size, warranting the necessity for larger-scale studies involving patients and controls. Future investigations will address these limitations by conducting follow-up studies with an increased number of subjects and employing a more specific study design, including subgroupings based on factors such as obesity or intrinsic/extrinsic AD. Comparative studies with established biomarkers, including cytokines, chemokines, and epidermal differential proteins, will be conducted to elucidate their relationships with IL-4 and IL-13. These endeavors aim to thoroughly evaluate the potential of IL-4 and IL-13 as novel biomarkers, providing a comprehensive understanding of their correlation with the disease activity and treatment response in AD.

AD is one of the most common chronic inflammatory skin conditions. The development of specific treatment options is difficult due to the complexity of the disease. AD is caused by a combination of genetic and environmental factors, which create a unique profile of each case, and this complexity of factors also provides an answer as to why there is currently no cure. Recent progress in understanding the pathophysiology of AD allowed the development of targeted therapies, rather than the classic concept of ”one-size-fits-all” previously used for AD treatment.

The onset of AD is characterized as a T-cell-mediated disease dominated by Th2 signaling in acute lesions, with a subsequent shift from Th2 to Th1 observed in the chronic state of the disease. Among the Th2 immune mediators, IL-4 and IL-13 have been demonstrated to play a key role in AD pathogenesis by stimulating sensory neurons directly. Furthermore, IL-4 and IL-13 polymorphisms had been shown to be genetically associated with AD [3,4]. Dupilumab, an IL-4 receptor alpha (IL-4Rα) antagonist, had been demonstrated to reduce the type 2 signature in the blood [19]. Thus, a decrease in the IL-4 and IL-13 serum levels is expected after Dupilumab administration.

In endeavors to identify biomarkers associated with the exacerbation or improvement of atopic dermatitis (AD), Ariëns et al. assessed twenty-one biomarkers linked to various AD pathways, including IL-4 and IL-13. Interestingly, they determined an increase in IL-4 and IL-13 upon treatment as a resultant side effect. It is assumed that blocking the IL-4 alpha receptor with Dupilumab might cause an increase in the unbound circulating IL-4 and IL-13 levels. This increase in IL-4 and IL-13 is most likely a temporary phenomenon since the long-term suppression of IL-4Rα will eventually lead to decreased production of IL-4 and IL-13 by the T cells [20]. The IL-22 serum levels correlated much better with the clinical improvement of the Dupilumab-treated patients. Several studies reported similar results, suggesting that IL-22 might be a potential reliable biomarker [21,22,23].

Recent findings have highlighted the role of various adipokines in the pathogenesis of various inflammatory skin diseases, including AD [24]. Among these biomarkers, adiponectin assumes a central role in the anti-inflammatory response by diminishing the production of TNF-α, IL-6, and interferon-gamma [25]. Seo et al. demonstrated that activation of the adiponectin pathway is expected to improve epidermal differentiation in AD [26], but still the precise role of adiponectin in AD pathogenesis remains incompletely understood. Nevertheless, one recent study tried to evaluate adiponectin as a biomarker for disease severity but failed to demonstrate a decrease in serum levels in successfully treated patients [27].

A recent review undertook an extensive analysis of AD biomarkers and concluded that thymus- and activation-regulated chemokine/C-C motif ligand 17 (TARC/CCL17) appears to be the most likely candidate for use as a reference biomarker, correlating with the disease severity in all patients, regardless of age [16]. However, He et al. demonstrated that these AD biomarkers are predominantly elevated in patients exhibiting moderate to severe and severe forms of the condition and are absent in mild disease [28]. Moreover, there have been reports that the TARC/CCL17 levels may vary between AD patients with similar disease severity scores [29], most likely due to the subjective component of these scores. Bodoor et al. also showed that the IL-4 and IL-13 serum levels were elevated but did not correlate with pruritus and disease severity in a cohort of 56 patients [30]. The study did not measure the dynamics of these biomarkers after any treatment.

A cohort of 125 patients underwent an investigation into additional biomarkers, including CD25/soluble interleukin (sIL)-2Rα, IL-31, and IL-36β. The study’s findings affirm that these biomarkers hold promise as predictive indicators for treatment response. The study observed a positive correlation between the efficacy of the treatment and an augmented value of these markers at the onset of therapy. This suggests that CD25/soluble interleukin (sIL)-2Rα, IL-31, and IL-36β could serve as valuable prognostic factors, offering insights into the anticipated treatment outcomes [31]. Paradoxically, as demonstrated in numerous studies [32,33,34], disease severity scores may improve even as IL-4 and IL-13 levels rise during Dupilumab therapy.

IL-4 and IL-13 share the same receptor, even though IL-13 can also bind to another receptor, which is regarded mainly as a decoy receptor [35]. Although blocking this common receptor for the two ILs brings significant clinical benefits in atopic dermatitis, this benefit is not found for all pathologies in which IL-4 and IL-13 intervene, as a recent study suggests that IL-4/IL-13’s anti-inflammatory properties could be useful for inflammatory arthritis treatment [36]. Additionally, in one of our own studies regarding the use of Dupilumab for AD treatment, we found that the use of this drug can lead to the progression of myeloproliferative disorders in patients in which the underlying disease is misdiagnosed as AD [37]. As we showed in the current study, inhibition of the receptor for IL-4 and IL-13 leads to an increased serum concentration of both ILs. An explanation for this phenomenon comes from the article recently published by Melo-Cardenas et al., in which the authors show that myeloproliferative disease progression is associated with increased IL-13 levels, whereas reducing IL-4 and IL-13 associates with reduced features of such disorders [38].

Interestingly, and in contradiction with other studies which suggest that a successful treatment for AD is associated with a reduction in IL-13 levels [39], our patients showed significant improvements in disease despite an increase in IL-13 levels. Other studies have indicated that the use of cyclosporine [40] as a sole treatment is linked to a reduction in IL-13 levels, as well as other therapeutic methods such as UVA exposure [41] or tacrolimus [42] used as sole treatment. Therefore, it appears that the inhibition of IL-13 alone could be beneficial for these patients in terms of disease amelioration and an improvement in quality of life. Nevertheless, the authors still conclude that a clear conclusion cannot be drawn in this regard [39]. The conclusions drawn from the aforementioned study are nevertheless supported by another article stating that IL-13 is significantly more highly expressed in AD compared to IL-4 at the level of the lesional skin but does not reference the serum levels of these ILs [43]. Regarding IL-4, treatment with Dupilumab induced its increase, as reported by Kamphuis et al., but only up to week 4; thereafter, it gradually declined until week 16, while the serum levels of IL-13 did not undergo significant changes [44]. 

One of the most intriguing studies addressing serum biomarkers of AD categorizes patients into four groups based on the prevalence of a specific category of markers. Out of the 146 patients included in the study, only 18.5% belonged to the category where IL-4 and IL-13, along with other Th2 and Th1 markers, predominated. Categorizing patients based on this endotype could be beneficial in assisting better decision-making for therapy selection for these patients [31]. 

Previous attempts at exploring classical biomarkers, such as Immunoglobulin E (IgE), yielded inconclusive results. While the IgE levels decreased in the majority of patients treated with Dupilumab, the limited sample size precludes definitive conclusions [45].

In our investigation, we assessed the potential of IL-4 and IL-13 as biomarkers for gauging the treatment response in patients with atopic dermatitis (AD) undergoing Dupilumab administration. However, it is crucial to acknowledge the limitations inherent in our study. Firstly, the inclusion of a limited number of patients hinders the establishment of a definitive pattern based on our findings. The small sample size introduces a degree of uncertainty, preventing us from drawing conclusive insights into the role of IL-4 and IL-13 as reliable biomarkers.

Secondly, our study did not observe a discernible decrease in the serum levels of IL-4 and IL-13. This finding raises questions about the utility of these cytokines as direct indicators of treatment response. Without a clear reduction in serum levels, it becomes challenging to link changes in IL-4 and IL-13 to the efficacy of Dupilumab in AD patients.

Thirdly, it is evident that relying solely on IL-4 and IL-13 serum levels may not be suitable as a comprehensive biomarker for evaluating treatment response. This is especially true in the context of Dupilumab treatment, where an increase in the values of these cytokines might be anticipated. In summary, while our study sheds light on the potential role of IL-4 and IL-13 as biomarkers, the limitations, such as the small sample size and lack of an observed serum level decrease, underscore the need for further research to elucidate their true significance in assessing the treatment response in AD patients undergoing Dupilumab administration. Major improvements in disease severity were also noticed for our patients, as shown in the decrease in the SCORAD and DLQI severity scores.

Nevertheless, our attempt aimed to establish an inverse correlation between IL values and severity scores. In other words, we sought to determine whether a discernible model could be formulated, demonstrating a proportional IL value growth magnitude that correlates with the extent of the reduction in the severity scores. The objective was to investigate whether, at the 3-month mark, an increase to a specific degree in IL values is linked to a corresponding decrease in severity scores. This exploration aimed to discern whether such a correlation could contribute to assessing therapeutic effectiveness. Unfortunately, despite our efforts, we were unable to identify a consistent pattern in the data. Moreover, increased levels of type 2 cytokines have been identified not only in AD but also in other conditions like eosinophilic dermatosis of hematological malignancy [46]. Further investigation could aim to assess the potential reduction in these cytokine levels using Dupilumab treatment in this uncommon condition, opening up avenues for valuable research.

### 4.2. Strengths and Limitations of the Study

This study possesses certain limitations that warrant acknowledgment. The limited sample size in our study restricts a definitive interpretation of the results, and the lack of a noticeable decrease in the IL-4 and IL-13 serum levels questions their direct utility as indicators of treatment response. Solely relying on the IL levels may be insufficient, underscoring the necessity for additional research to clarify their significance. Despite significant improvements in disease severity, efforts to establish a clear correlation between IL values and severity scores were inconclusive, illustrating the challenges in assessing therapeutic effectiveness in AD patients treated with Dupilumab. Nevertheless, it serves as an initial exploration for future studies, being among the first, to our knowledge, to assess IL-4 and IL-13 as biomarkers for atopic dermatitis.

## 5. Conclusions

The available clinical data and studies on IL-4 and IL-13 serum levels during Dupilumab treatment are limited. Furthermore, the existing data tend to be contradictory. However, recognizing the constraint imposed by our sample size, we acknowledge that future studies incorporating larger cohorts are essential to validate these biomarkers across diverse populations and enhance the generalizability of our results.

In this paper, we tried to follow the dynamics of IL-4 and IL-13 and possibly identify them as potential reliable biomarkers in AD. Taking into account our results, (1) IL levels are expected to increase; (2) the disease severity scores decrease; (3) the procedure for dosing these ILs is time-consuming and expensive compared to the usual bloodwork; (4) the results obtained do not influence subsequent therapeutic conduct—we conclude that the dosage of these ILs is not justified in day-to-day clinical practice. However, our study group was very small, insufficient for a clear conclusion, but still can represent a starting point for future studies. 

From a comprehensive standpoint, an ideal biomarker should possess 100% sensitivity and specificity and demonstrate accuracy, reproducibility, minimal invasiveness, clinical applicability, and easy measurability. Furthermore, when it comes to the management of AD, these biomarkers should reflect the disease severity, predict the course of disease, and correlate with the response to treatment. This could help in the management of patients with atopic dermatitis and the choice of the optimal treatment, especially since the symptomatology of atopic dermatitis is dominated by a very subjective component, pruritus, which can be experienced differently depending on the individual tolerability of each patient.

Considering the extensive array of potential biomarkers in AD, it is crucial to acknowledge that, as of yet, none of the identified biomarkers has been formally validated and universally accepted for routine clinical practice. The development of a new biomarker is a highly intricate and time-consuming process, encompassing discovery, validation, qualification, and eventual clinical utilization.

## Figures and Tables

**Table 1:** General characteristics of study subjects. SCORAD and DLQI scores are presented before treatment. * Mean value for the respective group.

Characteristic	Control	AD Patients
Age *	30.12	33.81
Sex	10 males (50%), 10 females (50%)	12 males (54.55%), 10 females (45.45%)
SCORAD *	-	47.64
DLQI *	-	19.41

**Table 2:** Summary of IL-4 and IL-13 levels in study subjects. Values presented are measured in pg/mL. Values are presented as mean ± standard deviation. * Statistical analysis was carried out using two-sample *t*-test.

Value before Treatment	Control	AD Patients	*p* Value *
IL-4	1.42 ± 0.61	9.51 ± 3.71	<0.001
IL-13	1.25 ± 0.78	15.55 ± 6.34	<0.001

**Table 3:** Summary of clinical and laboratory findings in the AD group. Values presented are measured in pg/mL. Values are presented as mean ± standard deviation. * Statistical analysis was carried out using a paired *t*-test.

Value	Before Treatment	After Treatment	*p* Value *
IL-4	9.51 ± 3.71	11.86 ± 4.19	<0.001
IL-13	15.55 ± 6.34	18.51 ± 6.10	<0.001
SCORAD	47.64 ± 8.09	6.45 ± 2.09	<0.001
DLQI	19.41 ± 3.57	2.73 ± 1.28	<0.001

## Data Availability

Data are contained within the article.

## Abbreviations

AD	Atopic dermatitis
IL(s)	Interleukin(s)
Th2	T helper cells type 2
Th22	T helper cells type 22
SCORAD	Scoring Atopic Dermatitis
DLQI	Dermatology Life Quality Index
POEM	Patient-Oriented Eczema Measure
ABS-A	Atopic Dermatitis-Specific Quality of Life
FDA	Food and Drug Administration
MDC/CCL22	Macrophage-derived chemokine
PARC/CCL18	Pulmonary and activation-regulated chemokine
CTACK/CCL27	Cutaneous T-cell-attracting chemokine
Eotaxin-3/CCL26	Eosinophil-attracting chemokine
IL-4Rα	Interleukin 4 receptor alpha
TNF-α	Tumor necrosis factor α
TARC/CCL17	Thymus- and activation-regulated chemokine/C-C motif ligand 17
IgE	Immunoglobulin E IgE

## References

[1] Eichenfield L.F., Tom W.L., Chamlin S.L., Feldman S.R., Hanifin J.M., Simpson E.L., Berger T.G., Bergman J.N., Cohen D.E., Cooper K.D. (2014). Guidelines of care for the management of atopic dermatitis: Section 1. Diagnosis and assessment of atopic dermatitis. J. Am. Acad. Dermatol..

[2] Richard M.A., Corgibet F., Beylot-Barry M., Barbaud A., Bodemer C., Chaussade V., D’Incan M., Joly P., Leccia M.T., Meurant J.M. (2018). Sex- and age-adjusted prevalence estimates of five chronic inflammatory skin diseases in France: Results of the «OBJECTIFS PEAU» study. J. Eur. Acad. Dermatol. Venereol..

[3] Brunner P.M., Guttman-Yassky E., Leung D.Y. (2017). The immunology of atopic dermatitis and its reversibility with broad-spectrum and targeted therapies. J. Allergy Clin. Immunol..

[4] Kader H.A., Azeem M., Jwayed S.A., Al-Shehhi A., Tabassum A., Ayoub M.A., Hetta H.F., Waheed Y., Iratni R., Al-Dhaheri A. (2021). Current Insights into Immunology and Novel Therapeutics of Atopic Dermatitis. Cells.

[5] Ferrucci S., Angileri L., Tavecchio S., Fumagalli S., Iurlo A., Cattaneo D., Marzano A.V., Maronese C.A. (2022). Elevation of peripheral blood eosinophils during dupilumab treatment for atopic dermatitis is associated with baseline comorbidities and development of facial redness dermatitis and ocular surface disease. J. Dermatolog Treat..

[6] Su Z., Zeng Y.P. (2023). Dupilumab-Associated Psoriasis and Psoriasiform Manifestations: A Scoping Review. Dermatology.

[7] Miniotti M., Lazzarin G., Ortoncelli M., Mastorino L., Ribero S., Leombruni P. (2022). Impact on health-related quality of life and symptoms of anxiety and depression after 32 weeks of Dupilumab treatment for moderate-to-severe atopic dermatitis. Dermatol. Ther..

[8] Mastorino L., Rosset F., Gelato F., Ortoncelli M., Cavaliere G., Quaglino P., Ribero S. (2022). Chronic Pruritus in Atopic Patients Treated with Dupilumab: Real Life Response and Related Parameters in 354 Patients. Pharmaceuticals.

[9] Taïeb A., Boralevi F., Seneschal J., Merhand S., Georgescu V., Taieb C., Ezzedine K. (2015). Atopic Dermatitis Burden Scale for Adults: Development and Validation of a New Assessment Tool. Acta Derm. Venereol..

[10] Ghosh D., Bernstein J.A., Khurana Hershey G.K., Rothenberg M.E., Mersha T.B. (2018). Leveraging Multilayered “Omics” Data for Atopic Dermatitis: A Road Map to Precision Medicine. Front. Immunol..

[11] Pavel A.B., Zhou L., Diaz A., Ungar B., Dan J., He H., Estrada Y.D., Xu H., Fernandes M., Renert-Yuval Y. (2020). The proteomic skin profile of moderate-to-severe atopic dermatitis patients shows an inflammatory signature. J. Am. Acad. Dermatol..

[12] Reiger M., Traidl-Hoffmann C., Neumann A.U. (2020). The skin microbiome as a clinical biomarker in atopic eczema: Promises, navigation, and pitfalls. J. Allergy Clin. Immunol..

[13] Rojahn T.B., Vorstandlechner V., Krausgruber T., Bauer W.M., Alkon N., Bangert C., Thaler F.M., Sadeghyar F., Fortelny N., Gernedl V. (2020). Single-cell transcriptomics combined with interstitial fluid proteomics defines cell type-specific immune regulation in atopic dermatitis. J. Allergy Clin. Immunol..

[14] FDA-NIH Biomarker Working Group (2016). BEST (Biomarkers, EndpointS, and Other Tools) Resource. https://www.ncbi.nlm.nih.gov/books/NBK326791/.

[15] Renert-Yuval Y., Thyssen J.P., Bissonnette R., Bieber T., Kabashima K., Hijnen D., Guttman-Yassky E. (2021). Biomarkers in atopic dermatitis-a review on behalf of the International Eczema Council. J. Allergy Clin. Immunol..

[16] Mastraftsi S., Vrioni G., Bakakis M., Nicolaidou E., Rigopoulos D., Stratigos A.J., Gregoriou S. (2022). Atopic Dermatitis: Striving for Reliable Biomarkers. J. Clin. Med..

[17] Hanifin J.M., Rajka G. (1980). Diagnostic features of atopic dermatitis. Acta Derm. Venereol..

[18] DUPIXENT® Dupilumab. https://www.dupixenthcp.com/atopicdermatitis/.

[19] Guttman-Yassky E., Bissonnette R., Ungar B., Suárez-Fariñas M., Ardeleanu M., Esaki H., Suprun M., Estrada Y., Xu H., Peng X. (2019). Dupilumab progressively improves systemic and cutaneous abnormalities in patients with atopic dermatitis. J. Allergy Clin. Immunol..

[20] Ariëns L.F.M., van der Schaft J., Bakker D.S., Balak D., Romeijn M.L.E., Kouwenhoven T., Kamsteeg M., Giovannone B., Drylewicz J., van Amerongen C.C.A. (2020). Dupilumab is very effective in a large cohort of difficult-to-treat adult atopic dermatitis patients: First clinical and biomarker results from the BioDay registry. Allergy.

[21] Nettis E., Ferrucci S.M., Ortoncelli M., Pellacani G., Foti C., Di Leo E., Patruno C., Rongioletti F., Argenziano G., Macchia L. (2022). Use of Dupilumab in 543 Adult Patients With Moderate-to-Severe Atopic Dermatitis: A Multicenter, Retrospective Study. J. Investig. Allergol. Clin. Immunol..

[22] Scibiorek M., Mthembu N., Mangali S., Ngomti A., Ikwegbue P., Brombacher F., Hadebe S. (2023). IL-4Rα signalling in B cells and T cells play differential roles in acute and chronic atopic dermatitis. Sci. Rep..

[23] Bakker D.S., Ariens L.F.M., Giovannone B., Hijnen D., Delemarre E.M., Knol E., Nierkens S., de Bruin-Weller M.S., Thijs J.L., Drylewicz J. (2020). EASI p-EASI: Predicting disease severity in atopic dermatitis patients treated with Dupilumab using a combination of serum biomarkers. Allergy.

[24] Kovács D., Fazekas F., Oláh A., Törőcsik D. (2020). Adipokines in the Skin and in Dermatological Diseases. Int. J. Mol. Sci..

[25] Fang H., Judd R.L. (2018). Adiponectin Regulation and Function. Compr. Physiol..

[26] Seo H.S., Seong K.H., Kim C.D., Seo S.J., Park B.C., Kim M.H., Hong S.P. (2019). Adiponectin Attenuates the Inflammation in Atopic Dermatitis-Like Reconstructed Human Epidermis. Ann. Dermatol..

[27] Lee S.H., Bae Y., Park Y.L. (2022). Clinical Implication of Serum Adiponectin Levels in Adult Patients with Atopic Dermatitis. J. Clin. Med..

[28] He H., Del Duca E., Diaz A., Kim H.J., Gay-Mimbrera J., Zhang N., Wu J., Beaziz J., Estrada Y., Krueger J.G. (2021). Mild atopic dermatitis lacks systemic inflammation and shows reduced nonlesional skin abnormalities. J. Allergy Clin. Immunol..

[29] Landheer J., de Bruin-Weller M., Boonacker C., Hijnen D., Bruijnzeel-Koomen C., Röckmann H. (2014). Utility of serum thymus and activation-regulated chemokine as a biomarker for monitoring of atopic dermatitis severity. J. Am. Acad. Dermatol..

[30] Bodoor K., Al-Qarqaz F., Heis L.A., Alfaqih M.A., Oweis A.O., Almomani R., Obeidat M.A. (2020). IL-33/13 Axis and IL-4/31 Axis Play Distinct Roles in Inflammatory Process and Itch in Psoriasis and Atopic Dermatitis. Clin. Cosmet. Investig. Dermatol..

[31] Wu Y., Gu C., Wang S., Yin H., Qiu Z., Luo Y., Li Z., Wang C., Yao X., Li W. (2023). Serum biomarker-based endotypes of atopic dermatitis in China and prediction for efficacy of Dupilumab. Br. J. Dermatol..

[32] Barbarot S., Wollenberg A., Silverberg J.I., Deleuran M., Pellacani G., Armario-Hita J.C., Chen Z., Shumel B., Eckert L., Gadkari A. (2022). Dupilumab provides rapid and sustained improvement in SCORAD outcomes in adults with moderate-to-severe atopic dermatitis: Combined results of four randomized phase 3 trials. J. Dermatolog Treat..

[33] Wollenberg A., Marcoux D., Silverberg J.I., Aoki V., Baselga E., Zhang H., Levit N.A., Taieb A., Rossi A.B. (2022). Dupilumab Provides Rapid and Sustained Improvement in SCORing Atopic Dermatitis Outcomes in Paediatric Patients with Atopic Dermatitis. Acta Derm. Venereol..

[34] Augustin M., Bauer A., Ertner K., von Kiedrowski R., Schenck F., Ramaker-Brunke J., Möller S., Fait A., Bastian M., Thaçi D. (2023). Dupilumab Demonstrates Rapid Onset of Action in Improving Signs, Symptoms and Quality of Life in Adults with Atopic Dermatitis. Dermatol. Ther..

[35] Brombacher F. (2000). The role of interleukin-13 in infectious diseases and allergy. Bioessays.

[36] Iwaszko M., Biały S., Bogunia-Kubik K. (2021). Significance of Interleukin (IL)-4 and IL-13 in Inflammatory Arthritis. Cells.

[37] Mitroi G.G., Stoica L.E., Mitroi G.F., Mitroi M.R., Tutunaru C.V., Ică O.M., Ianoși L.S. (2022). Atopic Dermatitis with Multiple Comorbidities Treated with Dupilumab. A Case Report and Review of the Literature Regarding the Safety of Dupilumab. Life.

[38] Melo-Cardenas J., Bezavada L., Crawford J.C., Gurbuxani S., Cotton A., Kang G., Gossett J., Marinaccio C., Weinberg R., Hoffman R. (2022). IL-13/IL-4 signaling contributes to fibrotic progression of the myeloproliferative neoplasms. Blood.

[39] May R.D., Fung M. (2015). Strategies targeting the IL-4/IL-13 axes in disease. Cytokine.

[40] Khattri S., Shemer A., Rozenblit M., Dhingra N., Czarnowicki T., Finney R., Gilleaudeau P., Sullivan-Whalen M., Zheng X., Xu H. (2014). Cyclosporine in patients with atopic dermatitis modulates activated inflammatory pathways and reverses epidermal pathology. J. Allergy Clin. Immunol..

[41] Gambichler T., Kreuter A., Tomi N.S., Othlinghaus N., Altmeyer P., Skrygan M. (2008). Gene expression of cytokines in atopic eczema before and after ultraviolet A1 phototherapy. Br. J. Dermatol..

[42] Simon D., Vassina E., Yousefi S., Kozlowski E., Braathen L.R., Simon H.U. (2004). Reduced dermal infiltration of cytokine-expressing inflammatory cells in atopic dermatitis after short-term topical tacrolimus treatment. J. Allergy Clin. Immunol..

[43] Bieber T. (2020). Interleukin-13: Targeting an underestimated cytokine in atopic dermatitis. Allergy.

[44] Kamphuis E., Boesjes C.M., Loman L., Bakker D.S., Poelhekken M., Zuithoff N.P.A., Kamsteeg M., Romeijn G.L.E., van Wijk F., de Bruin-Weller M.S. (2022). Dupilumab in daily practice for the treatment of pediatric atopic dermatitis: 28-week clinical and biomarker results from the BioDay registry. Pediatr. Allergy Immunol..

[45] Huang T.H., Chen Y.C., Lin S.Y., Chiu S.H., Yang T.T., Chiu L.W., Chen Y.Y., Lan C.E. (2019). Treatment of atopic dermatitis with Dupilumab in Taiwan: Dynamic changes of IgE levels as a potential response biomarker. Eur. J. Dermatol..

[46] Maronese C.A., Derlino F., Moltrasio C., Cattaneo D., Iurlo A., Marzano A.V. (2024). Neutrophilic and eosinophilic dermatoses associated with hematological malignancy. Front. Med..

